# A Tumor-Like Inverted Meckel’s Diverticulum: The Culprit Behind Adult Ileal Intussusception and a Diagnostic Pitfall

**DOI:** 10.7759/cureus.86090

**Published:** 2025-06-15

**Authors:** Hiroyuki Tokue, Hiroaki Yamada, Yoshito Tsushima

**Affiliations:** 1 Department of Diagnostic Radiology and Nuclear Medicine, Graduate School of Medicine, Gunma University, Maebashi, JPN

**Keywords:** acute abdominal pain, adult intussusception, ectopic gastric mucosa, hemorrhagic necrosis, meckel’s diverticulum

## Abstract

Meckel’s diverticulum (MD) is a rare but important differential diagnosis for adult intussusception, particularly when presenting with atypical imaging features, such as a tumor-like mass. Inverted MD with hemorrhagic necrosis is exceedingly rare and may mimic neoplastic lesions.

A 65-year-old man presented with acute abdominal pain and distension. He had experienced intermittent abdominal discomfort and a single episode of hematochezia two months prior to presentation. Abdominal computed tomography revealed an ileo-ileal intussusception with a round, high-attenuation mass at the lead point. Surgical resection was performed owing to concerns regarding malignancy. Intraoperatively, an inverted MD with hemorrhagic necrosis was identified. Histology confirmed ectopic gastric mucosa in the MD. The patient’s postoperative recovery was uneventful.

This case highlights a rare presentation of adult intussusception caused by an inverted necrotic MD mimicking an intraluminal tumor. Recognizing this condition is crucial, especially in patients with unexplained abdominal pain or prior gastrointestinal bleeding. MD should be considered in the differential diagnosis of distal ileal masses to ensure an accurate diagnosis and timely management.

## Introduction

Meckel’s diverticulum (MD) is a true congenital diverticulum resulting from incomplete obliteration of the vitelline duct during embryogenesis [1]. It is the most common congenital anomaly of the gastrointestinal tract, with an estimated prevalence of 1-2% in the general population [2]. Although most patients remain asymptomatic, MD can lead to various complications - including gastrointestinal bleeding, inflammation, intestinal obstruction, and intussusception - which may prompt a clinical diagnosis in both pediatric and adult patients [3]. Due to its nonspecific symptoms and variable imaging findings, MD can mimic several other conditions, including small bowel tumors, Crohn’s disease, intestinal duplication cysts, and appendicitis, depending on its location and complications. This wide range of differential diagnoses often delays accurate identification, particularly in adults [3].

The lifetime risk of developing complications related to MD is estimated at approximately 4%, with the likelihood of such complications decreasing with age [4]. While symptomatic MD is more frequently encountered in younger patients, inversion of the diverticulum leading to intussusception can also occur in older adults, albeit rarely [4]. This possibility must be considered, particularly when imaging reveals a tumor-like lesion in the distal ileum.

Herein, we report a rare case of adult ileal intussusception caused by an inverted MD that had undergone hemorrhagic necrosis and appeared as a tumor-like mass on computed tomography (CT). The lesion was indistinguishable from a neoplastic disease on imaging alone and required surgical resection for a definitive diagnosis. This case highlights the importance of including MD in the differential diagnosis of adult ileal intussusception, particularly in patients with a history of intermittent abdominal pain or unexplained lower gastrointestinal bleeding. Recognition of this condition is essential for timely and appropriate management.

## Case presentation

A 65-year-old man was referred to our hospital for evaluation and management of suspected intestinal intussusception. His medical history included a distal gastrectomy with Billroth I reconstruction, performed approximately 20 years earlier for gastric cancer. He was not taking any notable medications and had no family history of gastrointestinal disease.

The patient had been experiencing intermittent abdominal discomfort for the past 6 months. Two months prior to presentation, he passed a single episode of dark red stool. However, endoscopic examination at that time failed to identify a definitive source of bleeding, and no further investigations were performed as his symptoms resolved spontaneously.

On the day prior to admission, he developed dull, persistent epigastric pain at approximately 6:00 AM, which was followed by progressive abdominal distension several hours later. He denied having vomiting or diarrhea. He presented to a local clinic with worsening abdominal discomfort, prompting a referral to our institution for further evaluation.

On arrival, the patient was alert and oriented but appeared uncomfortable. His vital signs were stable: body temperature 36.8°C, pulse rate 92 beats per minute, blood pressure 142/90 mmHg, and oxygen saturation 96% on room air. Physical examination revealed a soft, flat abdomen with mild localized tenderness in the periumbilical region without rebound tenderness or guarding. The bowel sounds were mildly increased.

Laboratory tests on admission are summarized in Table 1. Notably, the patient showed mild hyponatremia, elevated total bilirubin, and an elevated D-dimer level, although inflammatory markers were only mildly increased. Although the patient appeared mildly uncomfortable, there were no signs of overt toxicity such as high-grade fever, hypotension, or altered mental status. His heart rate was mildly elevated (92 bpm), but he remained hemodynamically stable throughout.

**Table 1:** Laboratory findings on admission AST: aspartate transaminase; ALT: alanine transaminase; LDH: lactate dehydrogenase; ALP: alkaline phosphatase; γ-GTP: gamma-glutamyl transferase; BUN: blood urea nitrogen; PT-INR: prothrombin time: international normalized ratio; APTT: activated partial thromboplastin time

Parameter	Value	Reference Range
Total protein	7.6 g/dL	6.5–8.0 g/dL
Albumin	4.2 g/dL	3.8–5.3 g/dL
Total bilirubin	2.21 mg/dL	0.2–1.2 mg/dL
Direct bilirubin	0.47 mg/dL	0.0–0.4 mg/dL
AST (GOT)	21 U/L	13–30 U/L
ALT (GPT)	15 U/L	7–23 U/L
LDH	231 U/L	120–245 U/L
ALP	108 U/L	38–113 U/L
γ-GTP	46 U/L	10–47 U/L
BUN	15.7 mg/dL	8–20 mg/dL
Creatinine	0.77 mg/dL	0.6–1.1 mg/dL
Na	132 mEq/L	136–145 mEq/L
Cl	92 mEq/L	98–107 mEq/L
K	4.5 mEq/L	3.6–5.0 mEq/L
Ca	9.8 mg/dL	8.5–10.2 mg/dL
C-reactive protein (CRP)	0.57 mg/dL	<0.3 mg/dL
Glucose	124 mg/dL	70–110 mg/dL (fasting)
HbA1c (NGSP)	6.50%	4.6–6.2%
WBC	9500 /μL	4000–9000 /μL
RBC	4.16 × 10⁶ /μL	4.2–5.7 ×10⁶ /μL (male)
Hemoglobin	14.1 g/dL	13.5–17.6 g/dL (male)
Platelets	21.1 × 10⁴ /μL	15–35 ×10⁴ /μL
Prothrombin activity (PT)	122.70%	70–130%
PT-INR	0.89	0.85–1.15
APTT	31.5 sec	25–35 sec
D-dimer	2.0 μg/mL	<1.0 μg/mL

**Figure 1:**
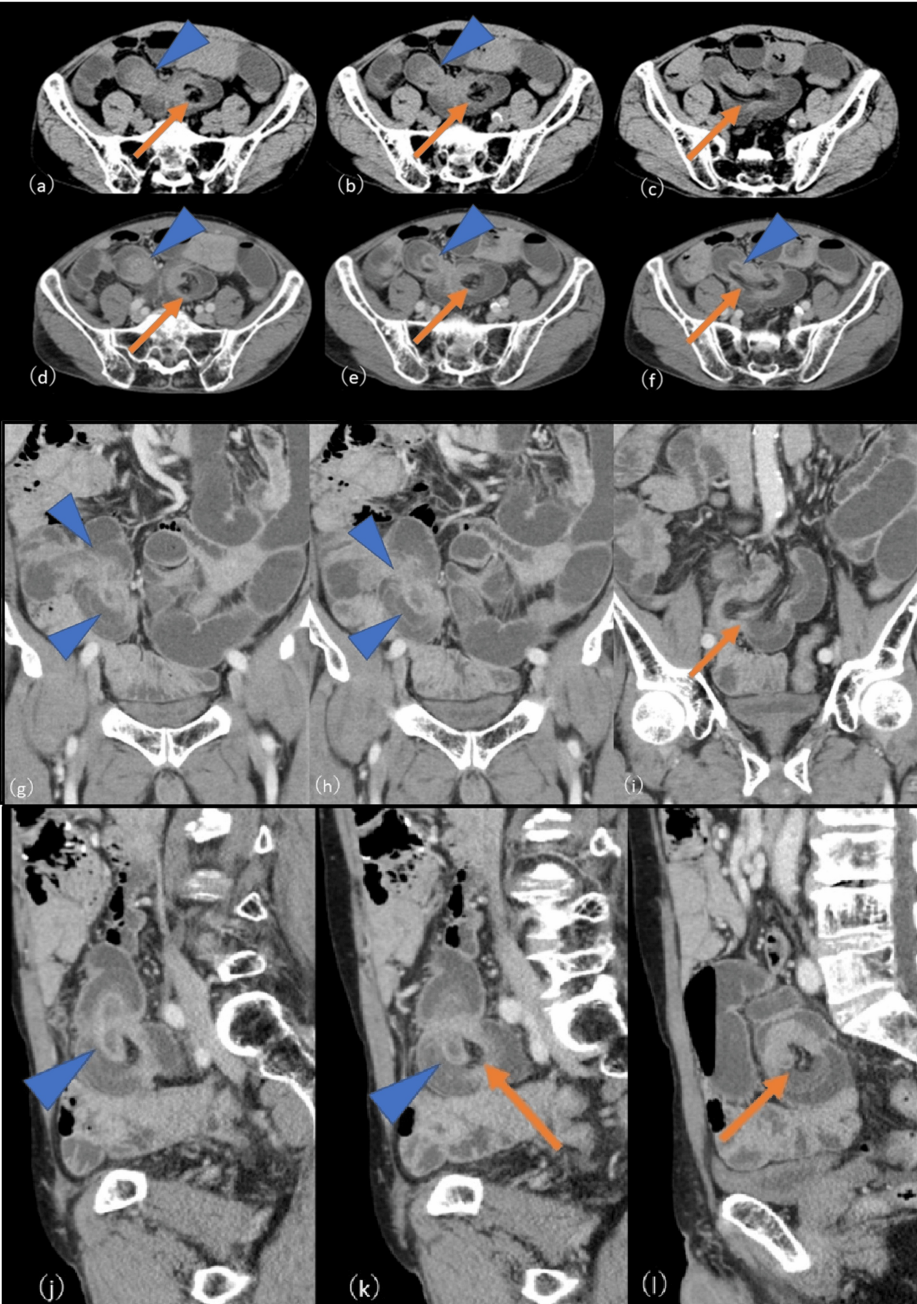
(a-c): Unenhanced CT (axial), (d-f): Contrast-enhanced CT (axial), (g-i): Contrast-enhanced CT (coronal), (j-l): Contrast-enhanced CT (sagittal) Abdominal CT revealing ileo-ileal intussusception in the distal ileum, with mesenteric fat trapped between the bowel loops (arrows). A high-attenuation, round mass is visualized at the leading edge of the intussusception, which is continuous with a tubular structure and exhibiting poor contrast enhancement (arrowheads).

Abdominal CT revealed an ileo-ileal intussusception in the distal ileum, with mesenteric fat trapped between the bowel loops. At the leading edge of the intussusception, a high-attenuation, round mass continuous with a tubular structure was visualized, showing poor contrast enhancement (Figures 1a-1l).

Proximal to the intussusception, dilatation of the small bowel was observed. Based on the imaging findings, the differential diagnoses included hemorrhagic or necrotic neoplasm, inverted necrotic diverticulum, or intestinal duplication.

**Figure 2:**
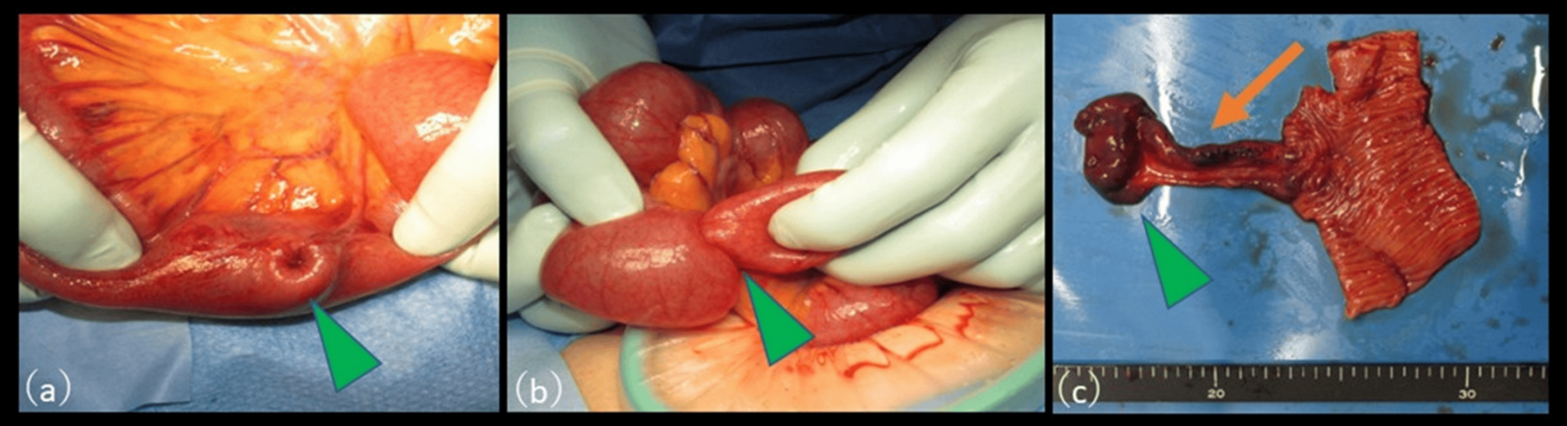
Intraoperative findings and surgical specimen (a) Intraoperatively, a segment of the ileum located approximately 100 cm proximal to the ileocecal valve is invaginated (arrowhead). (b) A firm intraluminal mass approximately 4 cm in diameter is palpable distal to the intussusceptum, and manual reduction was unsuccessful (arrowhead). (c) Gross examination of the resected specimen revealed a 10-cm-long true diverticulum (arrow) that was inverted into the intestinal lumen and appeared as a tumor-like mass (arrowhead).

Given the likelihood of intussusception secondary to an underlying organic lesion, emergency laparotomy was performed. Intraoperatively, a segment of the ileum located approximately 100 cm proximal to the ileocecal valve was invaginated (Figure 2a). A firm intraluminal mass approximately 4 cm in diameter was palpable distal to the intussusceptum, and manual reduction was unsuccessful (Figure 2b). A malignant neoplasm could not be excluded; thus, a 20-cm segment of the affected ileum, including the mass, was resected, and end-to-end anastomosis was performed.

Gross examination of the resected specimen revealed a 10-cm-long true diverticulum, which had inverted into the intestinal lumen and appeared as a tumor-like mass (Figure 2c).

**Figure 3:**
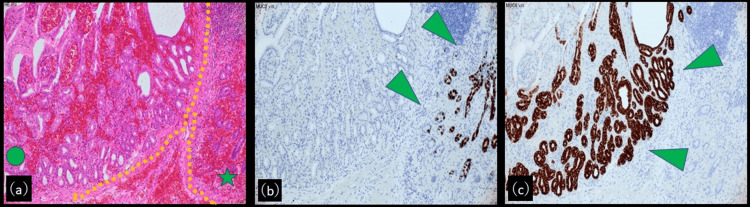
Pathological findings (a) Histologically, the diverticulum shows extensive hemorrhage and necrosis (Hematoxylin and eosin staining, original magnification 10×.). Areas of ectopic gastric mucosa (circle). Areas of small intestinal mucosa (star). (b) The small intestinal mucosa is positive for mucin2 (MUC2) within the diverticulum (original magnification: 10×). (c) The ectopic gastric glandular epithelium within the diverticulum is positive for MUC6 (original magnification: 10×).

The surface of the diverticulum showed extensive hemorrhage and necrosis, and the mucosal surface was irregular. Histopathological examination revealed the small intestinal mucosa positive for mucin2 (MUC2) and ectopic gastric glandular epithelium positive for MUC6 within the diverticulum (Figures 3a-3c).

Most diverticular tissue showed hemorrhagic necrosis. No epithelial atypia or neoplastic transformation was found, and the surgical margins contained normal small intestinal mucosa. The final diagnosis was MD with hemorrhagic necrosis.

The postoperative course was uneventful. Oral intake was resumed on postoperative day 5, and the patient was discharged on day 12. At the 6-month follow-up visit, the patient remained asymptomatic with no recurrence of gastrointestinal bleeding.

## Discussion

Adult intussusception is a rare clinical entity and, unlike in pediatric populations, is most often associated with an underlying structural abnormality [5]. In small bowel intussusception, common causes include benign tumors (such as lipomas or hamartomas), malignant neoplasms, diverticula, and postoperative adhesions [5]. In the present case, an MD that had undergone hemorrhagic necrosis and assumed a tumor-like appearance served as the lead point for the intussusception.

MD arises from incomplete obliteration of the vitelline duct during embryological development [6]. It is typically located at the antimesenteric border of the ileum, approximately 50-100 cm proximal to the ileocecal valve [7]. Most adult MD patients are asymptomatic. In a large retrospective study of 1,476 patients with MD, only 16% were symptomatic [8]. The clinical presentation of MD varies depending on associated complications. According to a previous report, obstruction is the most common presentation in children aged <11 years (40%), whereas bleeding is predominant in older children and adults (38%). Other frequent manifestations in adults include intestinal obstruction (34%) and diverticulitis (28%) [9].

Ectopic mucosa - most commonly gastric or pancreatic tissue - is found in many cases of MD [10]. In particular, the gastric mucosa is capable of acid secretion, leading to ulceration, inflammation, and bleeding. In the present case, immunohistochemical analysis demonstrated an MUC6-positive gastric-type epithelium within the diverticulum, supporting the presence of ectopic gastric mucosa. This ectopic tissue likely contributed to ulcer formation, local hemorrhage, and necrosis, ultimately resulting in inversion of the diverticulum and intussusception.

Notably, the patient had a prior episode of dark red stool approximately 2 months before presentation. Although no bleeding source was identified at that time, the subsequent histological findings of the ectopic gastric mucosa suggested that earlier bleeding may have originated from a peptic ulcer within the MD. Although gastrointestinal bleeding due to MD is more frequently reported in children, this case underscores the fact that similar mechanisms can also occur in adults and should not be overlooked.

Thus, in adult patients presenting with unexplained intermittent abdominal pain or occult lower gastrointestinal bleeding, MD should be considered a possible underlying cause. Furthermore, MD is not merely a structural anomaly leading to mechanical complications, such as intussusception; it may also present with ulcer-related bleeding or pain, reflecting its multifaceted clinical behavior.

The proposed mechanism of intussusception in cases of an inverted MD involves traction of the diverticular tip, which often contains ectopic mucosa and associated fat, into the bowel lumen [11]. Invagination may be exacerbated by increased intraluminal pressure and peristalsis, ultimately leading to full intussusception [11]. In our case, the diverticulum became necrotic and mass-like, making its radiological differentiation from a neoplasm particularly challenging.

Although MD is a rare cause of adult intussusception, several studies have reported its occurrence in 3-5% of small bowel intussusception cases [1,4,12,13]. Therefore, despite its rarity, MD should always be included in the differential diagnosis, particularly in cases involving the distal ileum.

The role of preoperative diagnostic imaging was crucial in this case. Contrast-enhanced CT revealed an ileo-ileal intussusception with a well-defined intraluminal mass at the lead point, supporting the diagnosis of a mechanical obstruction and prompting surgical intervention. However, the imaging features-particularly the mass-like appearance and poor contrast enhancement-closely mimicked a neoplasm, and a definitive diagnosis could not be established preoperatively. While contrast-enhanced CT remains the first-line modality for evaluating suspected intussusception due to its ability to detect both intra- and extra-luminal pathologies, other imaging techniques such as small bowel barium and ultrasound may provide complementary information. However, in acute or emergency settings, CT is generally preferred for its rapid acquisition and superior anatomical detail [14].

## Conclusions

In conclusion, this case illustrates a rare but clinically significant presentation of adult ileal intussusception caused by an inverted MD with hemorrhagic necrosis. The lesion closely mimicked a neoplastic mass on imaging, and the patient’s prior episode of hematochezia was retrospectively attributed to ulceration from ectopic gastric mucosa within the diverticulum. This underscores the protean manifestations of MD and the diagnostic challenge it can pose, particularly in adult patients.

Given its potential to present with tumor-like features, inverted MD should be considered in the differential diagnosis of adult intussusception. While contrast-enhanced CT is often the first-line imaging modality, it may not reliably distinguish between benign and malignant lead points. Surgical exploration remains essential for definitive diagnosis and appropriate treatment. Early recognition and timely intervention are critical to prevent complications and improve clinical outcomes.
